# Supplementary material for: Non-random mating patterns within and across education and mental and somatic health
Source: Nat Commun. 2024 Dec 3;15:10505. doi: 10.1038/s41467-024-54966-9 (PMC11615381; doi:10.1038/s41467-024-54966-9)
Supplement: Supplementary file 1 — Supplementary information [file 41467_2024_54966_MOESM1_ESM.pdf]

SUPPLEMENTARY INFORMATION

Non-random mating patterns within and across education and mental and somatic health

FA Torvik, HF Sunde, R Cheesman, NH Eftedal, MC Keller, E Ystrom, EM Eilertsen

Corresponding author: Fartein Ask Torvik, fartein.ask.torvik@fhi.no

Table of contents

Supplemental information for prospective analyses
Supplemental Figure S1. Within person correlations S2

Supplemental information for prospective analyses with odds ratios
Supplemental Figure S2. Within person associations as odds ratios S3
Supplemental Figure S3. Within and across-trait partner associations as odds ratios S4
Supplemental Table S1. Results from logistic regression among partners and in-laws S5

Supplemental information for cross-sectional analyses
Supplemental Table S2. Prevalence rates S6
Supplemental Table S3. Correlations between relatives S7
Supplemental Figure S4. Prevalence rates among affected and unaffected partners S8
Supplemental Figure S5. Correlations between partners with various adjustments S9
Supplemental Figure S6. Within person correlations S10
Supplemental Figure S7. Partner correlations adjusted for age S11
Supplemental Figure S8. Partner correlations adjusted for age and GPA S12
Supplemental Figure S9. Partner correlations adjusted for age and EA S13

Supplemental information for methods
Supplemental Script S1. Calculation of the in-law inflation factor (IIF) S14
Supplemental Script S2. Potential bias in tetrachoric correlations for skewed variables S15
Supplemental Script S3. Potential bias in the product of tetrachoric correlations S16

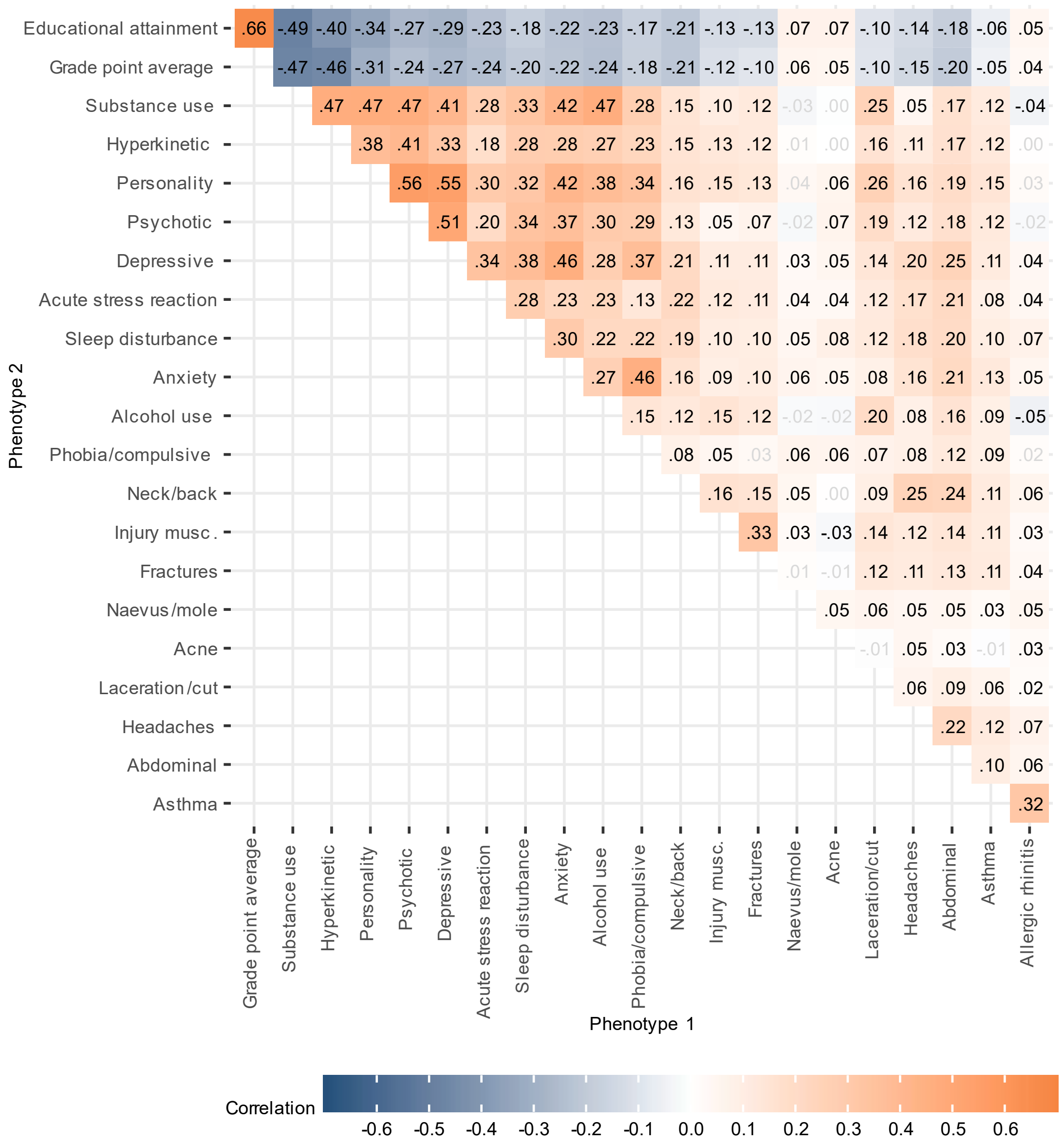

Supplemental Figure S1. Prospective within person correlations. Within person correlations for educational outcomes, 10 mental health conditions, and 10 somatic health conditions, 10 to 5 years before first child (n=187,926). Adjusted for age and sex. We tested whether the correlations differ from zero using two-sided z-tests based on the estimated correlations and their standard errors provided by OpenMx. Significant correlations (p < 0.05 after Benjamini-Hochberg adjustment) are shown in black. Exact p-values are provided in the Source Data file.

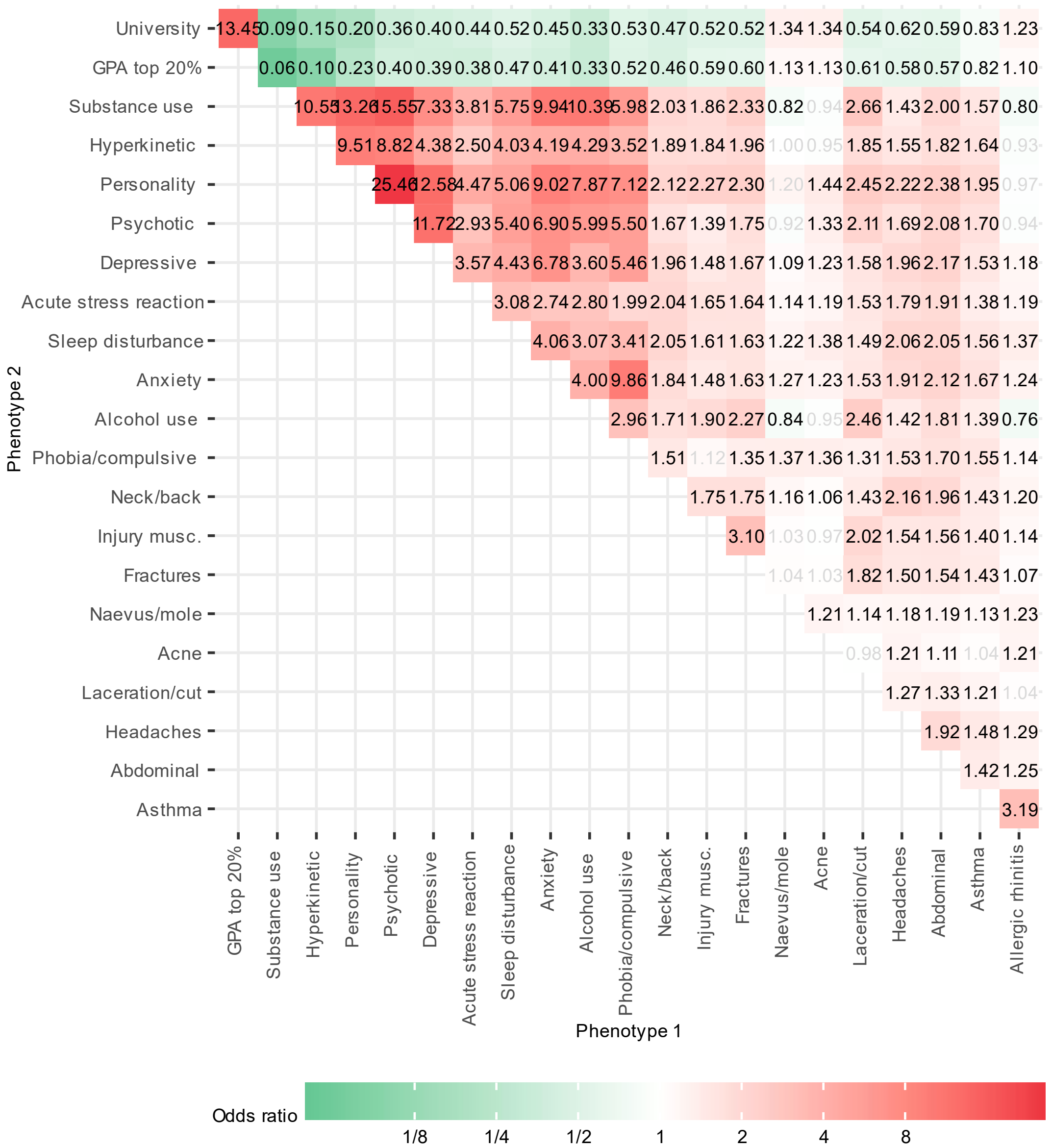

Supplemental Figure S2. Prospective within person associations as odds ratios. Odds rations for within person associations between educational outcomes, 10 mental health conditions, and 10 somatic health conditions, 10 to 5 years before first child (n=187,926). Adjusted for age and sex. Odds ratios and their standard errors were estimated using the `glm` function in R. Significant associations (two-sided p < 0.05 after Benjamini-Hochberg adjustment) are shown in black. Exact p-values are provided in the Source Data file.

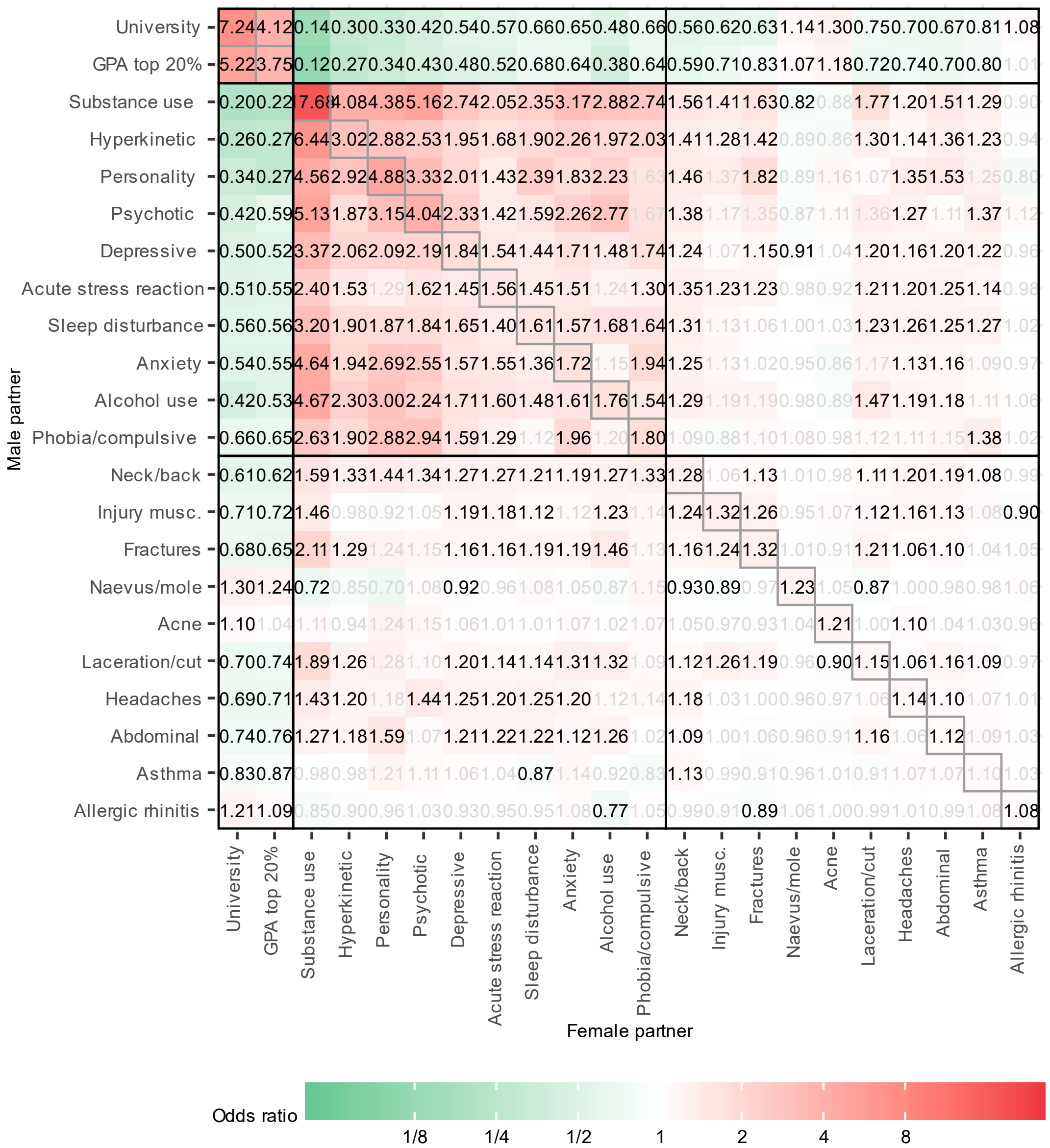

Supplemental Figure S3. Prospective within and across-trait partner associations as odds ratios. Odds ratios for within and across-trait partner associations between educational outcomes, 10 mental health conditions, and 10 somatic health conditions, 10 to 5 years before first child, all adjusted for age (n=93,963 couples). Odds ratios and their standard errors were estimated using the `glm` function in R. Significant associations (two-sided p < 0.05 after Benjamini-Hochberg adjustment) are shown in black. Exact p-values are provided in the Source Data file.

Supplemental Table S1. Results from multiple binary logistic regressions of each of the educational outcomes and the 10 mental and 10 somatic health conditions on the corresponding traits in partners, siblings, and in-laws. Health conditions were measured 10 to 5 years before a couple had their first child. Results expressed as adjusted odds ratios (OR), including 95% confidence intervals. Significant adjusted associations with siblings-in-law indicate deviations from direct assortment (n=93,963 couples; n=156,335 siblings; n=156,335 in-laws).

	OR(partner)	OR(sibling)	OR(in-law)
University education	5.35 [5.11, 5.59]	3.58 [3.44, 3.73]	1.76 [1.69, 1.83]
GPA among top 20%	2.76 [2.55, 3.00]	4.57 [4.22, 4.94]	1.54 [1.42, 1.68]
Substance use disorders	16.07 [12.83, 20.12]	4.91 [3.61, 6.68]	3.74 [2.75, 5.08]
Hyperkinetic disorder	2.73 [2.17, 3.43]	6.40 [5.37, 7.62]	1.37 [1.03, 1.81]
Personality disorder	1.99 [0.49, 8.06]	3.23 [1.19, 8.74]	3.39 [1.25, 9.18]
Psychotic disorders	2.91 [1.53, 5.51]	3.41 [2.08, 5.59]	0.96 [0.39, 2.32]
Depressive disorder	1.77 [1.62, 1.94]	2.36 [2.17, 2.56]	1.27 [1.15, 1.39]
Acute stress reaction	1.46 [1.31, 1.63]	2.72 [2.48, 2.98]	1.13 [1.01, 1.26]
Sleep disturbance	1.58 [1.38, 1.82]	1.89 [1.66, 2.15]	1.05 [0.90, 1.24]
Anxiety disorder	1.59 [1.29, 1.96]	2.68 [2.28, 3.16]	1.18 [0.94, 1.47]
Alcohol use disorders	1.69 [1.16, 2.46]	2.04 [1.39, 2.98]	1.41 [0.91, 2.19]
Phobia/compulsive disorder	1.73 [1.10, 2.71]	2.17 [1.49, 3.15]	1.38 [0.85, 2.25]
Neck/back symptom	1.25 [1.19, 1.31]	1.49 [1.42, 1.57]	1.14 [1.08, 1.20]
Injury musculoskeletal	1.29 [1.17, 1.41]	1.63 [1.49, 1.79]	1.13 [1.03, 1.25]
Fractures	1.27 [1.14, 1.42]	1.42 [1.27, 1.58]	1.19 [1.06, 1.33]
Naevus/mole	1.18 [1.09, 1.27]	1.61 [1.50, 1.73]	1.06 [0.98, 1.14]
Acne	1.14 [1.00, 1.30]	2.33 [2.10, 2.59]	0.96 [0.83, 1.11]
Laceration/cut	1.18 [1.08, 1.28]	1.21 [1.11, 1.32]	1.04 [0.96, 1.14]
Headaches	1.11 [1.03, 1.19]	1.49 [1.39, 1.59]	1.05 [0.98, 1.12]
Abdominal pain	1.11 [1.04, 1.18]	1.41 [1.33, 1.50]	1.06 [1.00, 1.13]
Asthma	1.12 [0.99, 1.27]	2.50 [2.28, 2.75]	1.04 [0.92, 1.18]
Allergic rhinitis	1.09 [1.00, 1.18]	2.03 [1.89, 2.18]	1.02 [0.94, 1.12]

Supplemental Table S2. List of ICPC-2 codes for the mental and somatic health condition, prevalence in the sample (including education), and prevalence among partners and relatives of affected individuals, measured cross sectionally in 2015-2019 (n=187,926 focal individuals; n=156,335 siblings; n=156,335 in-laws).

		Index		Partner of affected		Sibling of affected		In-law of affected	
Variable	ICPC-2 codes	n	%	n	%	n	%	n	%
University education		93,303	49.84	64,110	68.89	48,682	61.23	46,001	58.16
GPA among top 20%		26,824	20.15	8,058	37.43	7,654	42.38	5,085	32.38
Substance use disorders	P18, P19	2,899	1.54	724	24.97	163	8.22	143	6.76
Hyperkinetic disorder	P81	5,216	2.78	618	11.85	542	14.03	223	5.60
Personality disorder	P80	1,652	0.88	64	3.87	53	4.39	25	1.99
Psychotic disorders	P72, P98, P73	1,965	1.05	80	4.07	95	6.26	29	1.88
Depressive disorder	P76	21,811	11.61	4,126	18.92	2,966	17.59	2,352	13.47
Acute stress reaction	P02	24,996	13.30	5,442	21.77	3,320	16.81	2,564	12.67
Sleep disturbance	P06	16,807	8.94	2,424	14.42	1,619	12.17	1,312	9.63
Anxiety disorder	P74	8,202	4.36	648	7.90	560	8.83	353	5.34
Alcohol use disorders	P15, P16	2,505	1.33	158	6.31	91	4.83	64	3.29
Phobia/compulsive disorder	P79	3,702	1.97	146	3.94	128	4.44	93	3.12
Neck/back symptom/complaint	L01, L02, L03	44,851	23.87	12,446	27.75	8,342	23.27	7,633	20.90
Injury musculoskeletal	L81	12,271	6.53	1,094	8.92	911	9.16	800	7.96
Fractures	L72, L73, L74, L75, L76	10,641	5.66	764	7.18	694	8.01	556	6.40
Naevus/mole	S82	29,454	15.67	5,030	17.08	4,535	18.26	3,614	14.47
Acne	S96	7,721	4.11	326	4.22	638	10.00	356	5.53
Laceration/cut	S18	15,377	8.18	1,388	9.03	1,299	10.15	1,152	9.11
Headaches	N89, N90, N01, N95	32,011	17.03	5,148	16.08	4,808	18.59	4,136	15.64
Abdominal pain/cramps general	D01	36,242	19.29	6,878	18.98	5,427	18.73	4,809	16.17
Asthma	R96	11,415	6.07	800	7.01	1,069	11.51	598	6.35
Allergic rhinitis	R97	23,335	12.42	3,224	13.82	3,447	17.81	2,222	11.38

Supplemental Table S3. Correlations between relatives in educational outcomes and 10 mental and 10 somatic health conditions measured cross-sectionally in 2015-2019, including 95% confidence intervals, along with tests of deviations from direct assortment. Adjusted for sex and year of birth (n=93,963 couples; n=156,335 siblings; n=156,335 in-laws). Source data are provided in the source data file.

Variable	*r*(partner)	*r*(sibling)	*r*(inlaw)	Inlaw inflation factor (IIF)*	Deviation from direct assortment, p-value**
Educational attainment	0.48 [0.47, 0.48]	0.40 [0.40, 0.40]	0.29 [0.28, 0.29]	1.50	<1.00e-99
Grade point average	0.42 [0.42, 0.43]	0.52 [0.52, 0.53]	0.29 [0.29, 0.30]	1.33	<1.00e-99
Substance use disorders	0.67 [0.65, 0.70]	0.32 [0.29, 0.36]	0.27 [0.24, 0.31]	1.25	0.006
Hyperkinetic disorder	0.33 [0.30, 0.36]	0.38 [0.36, 0.40]	0.13 [0.10, 0.16]	1.03	0.805
Personality disorder	0.24 [0.17, 0.30]	0.24 [0.19, 0.29]	0.10 [0.05, 0.16]	1.83	0.234
Psychotic disorders	0.25 [0.19, 0.31]	0.23 [0.19, 0.27]	0.02 [-0.04, 0.07]	0.31	0.236
Depressive disorder	0.25 [0.23, 0.27]	0.17 [0.16, 0.19]	0.07 [0.05, 0.08]	1.57	0.002
Acute stress reaction	0.25 [0.23, 0.26]	0.19 [0.18, 0.20]	0.06 [0.05, 0.08]	1.36	0.023
Sleep disturbance	0.17 [0.15, 0.19]	0.13 [0.11, 0.14]	0.05 [0.03, 0.06]	2.26	0.003
Anxiety disorder	0.17 [0.14, 0.20]	0.18 [0.16, 0.20]	0.05 [0.02, 0.07]	1.53	0.280
Alcohol use disorders	0.28 [0.23, 0.33]	0.20 [0.15, 0.24]	0.11 [0.07, 0.16]	2.09	0.023
Phobia/compulsive disorder	0.14 [0.09, 0.18]	0.13 [0.10, 0.17]	0.07 [0.03, 0.11]	3.68	0.026
Neck/back symptom	0.12 [0.11, 0.13]	0.11 [0.10, 0.12]	0.05 [0.04, 0.06]	3.35	4.50e-09
Injury musculoskeletal	0.09 [0.07, 0.12]	0.08 [0.07, 0.10]	0.04 [0.02, 0.06]	4.71	0.006
Fractures	0.07 [0.04, 0.10]	0.08 [0.06, 0.10]	0.02 [-0.00, 0.04]	2.70	0.387
Naevus/mole	0.07 [0.06, 0.09]	0.13 [0.11, 0.14]	0.01 [0.00, 0.03]	1.58	0.403
Acne	0.04 [0.01, 0.08]	0.16 [0.14, 0.19]	0.02 [-0.01, 0.05]	2.95	0.355
Laceration/cut	0.06 [0.04, 0.08]	0.08 [0.06, 0.09]	0.04 [0.02, 0.05]	7.79	0.001
Headaches	0.06 [0.04, 0.07]	0.11 [0.10, 0.12]	0.03 [0.01, 0.04]	3.95	0.006
Abdominal pain	0.09 [0.07, 0.10]	0.09 [0.08, 0.10]	0.02 [0.01, 0.03]	2.97	0.021
Asthma	0.04 [0.01, 0.06]	0.19 [0.18, 0.21]	0.02 [-0.00, 0.04]	2.67	0.301
Allergic rhinitis	0.04 [0.02, 0.06]	0.18 [0.17, 0.19]	-0.00 [-0.01, 0.01]	-0.07	0.330

Notes: * IIF is expected to equal 1.00 under direct assortment. The cross-sectional partner correlations can be influenced by convergence, generally reducing the IIF. This may cancel out effects of indirect assortment and social stratification, which generally increases IIF, thereby limiting the interpretability of IIF among established couples. ** The p-values result from likelihood-ratio tests (1 degree of freedom) comparing a constrained model, where the in-law correlation is the product of partner and sibling correlations, to an unconstrained model with independent estimates for each relationship type. The p-values are adjusted for false discovery rate using the Benjamini-Hochberg method. A low p-value signifies a poor fit for direct assortment.

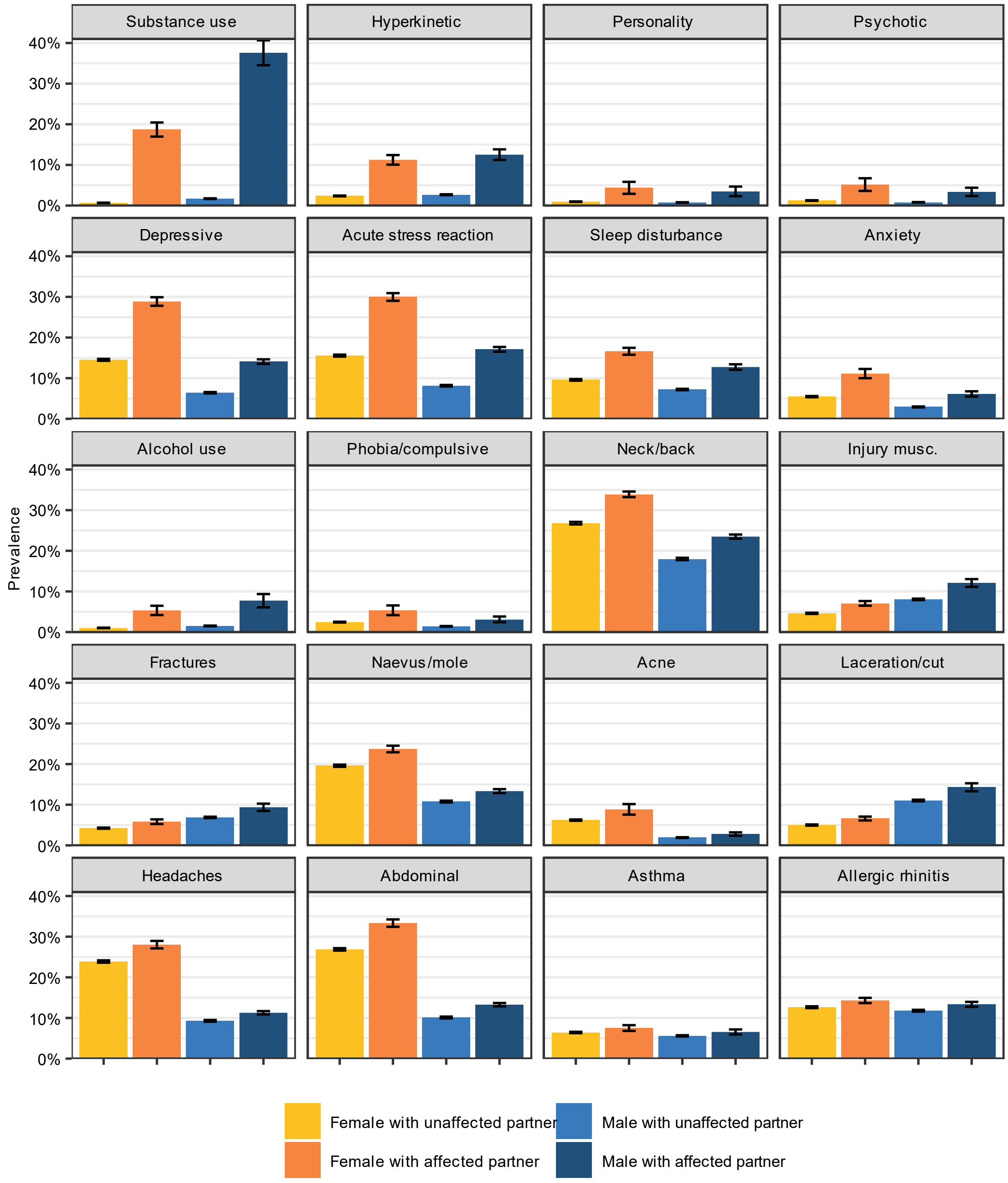

Supplemental Figure S4. Prevalence rates among affected and unaffected partners. Prevalence of 10 mental and 10 somatic health conditions among males (n=93,963) and females (n=93,963) with unaffected and affected partners, measured cross sectionally in 2015-2019. Data are presented as the proportions of diagnosed individuals, with error bars indicating 95% confidence intervals. Source data are provided in the source data file.

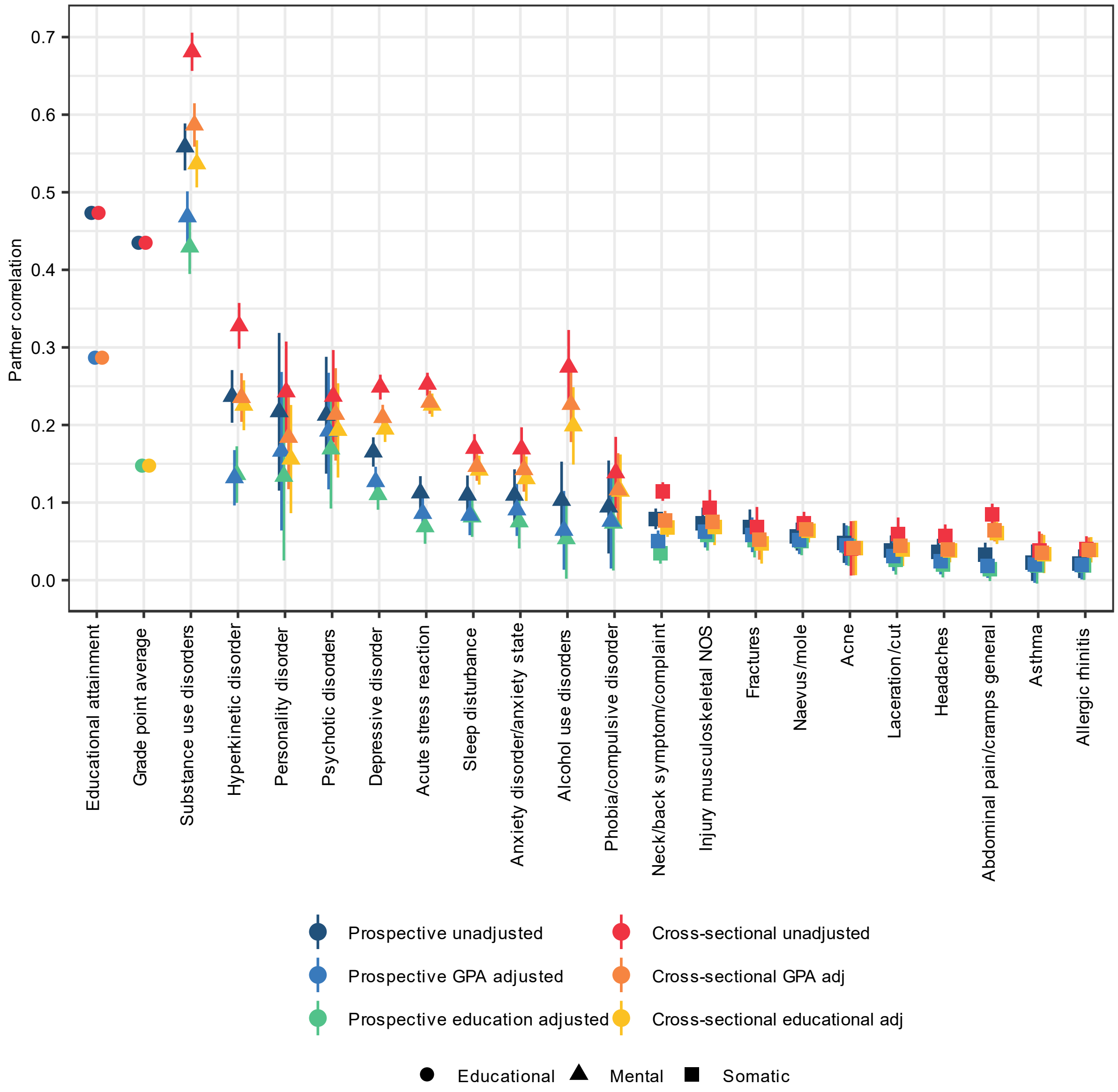

Supplemental Figure S5. Correlations between partners with various adjustments. Correlations between female and male partners for educational outcomes and 10 mental health and 10 somatic health phenotypes 10 to 5 years before they had their first child and cross-sectionally in 2015-2019 (n=93,963 couples). Including adjustment for educational attainment and grade point average in both assessments. Source data are provided in the source data file.

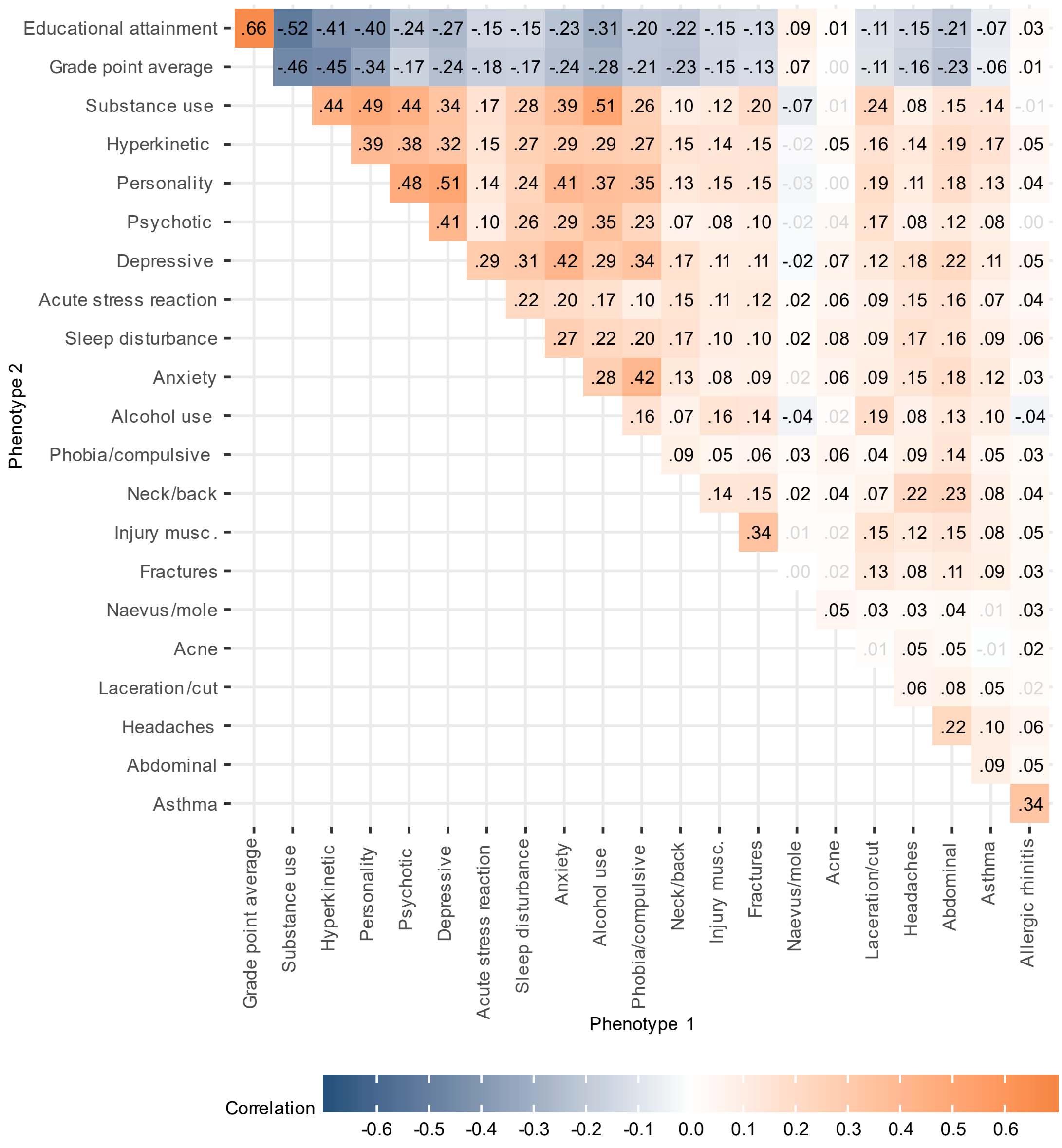

Supplemental Figure S6. Cross-sectional within person correlations. Within person correlations for educational outcomes, 10 mental health conditions, and 10 somatic health conditions, measured cross sectionally in 2015-2019 (n=187,926). Adjusted for age and sex. We tested whether the correlations differ from zero using two-sided z-tests based on the estimated correlations and their standard errors provided by OpenMx. Significant correlations (p < 0.05 after Benjamini-Hochberg adjustment) are shown in black. Exact p-values are provided in the Source Data file.

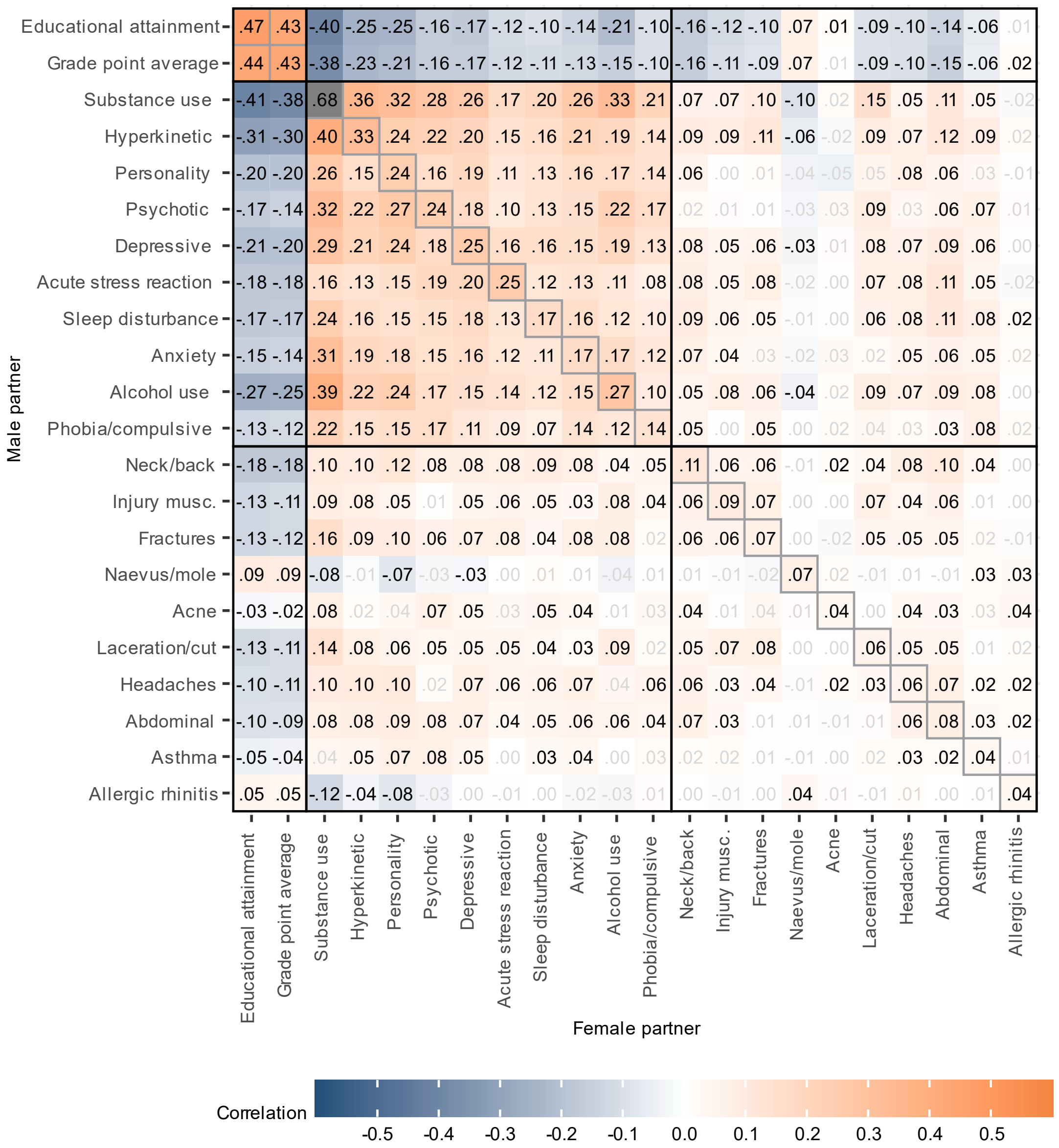

Supplemental Figure S7. Cross-sectional partner correlations. Within and across-trait partner correlations for educational outcomes, 10 mental health conditions, and 10 somatic health conditions, measured cross sectionally in 2015-2019 (n=93,963 couples). Adjusted for age. We tested whether the correlations differ from zero using two-sided z-tests based on the estimated correlations and their standard errors provided by OpenMx. Significant correlations (p < 0.05 after Benjamini-Hochberg adjustment) are shown in black. Exact p-values are provided in the Source Data file.

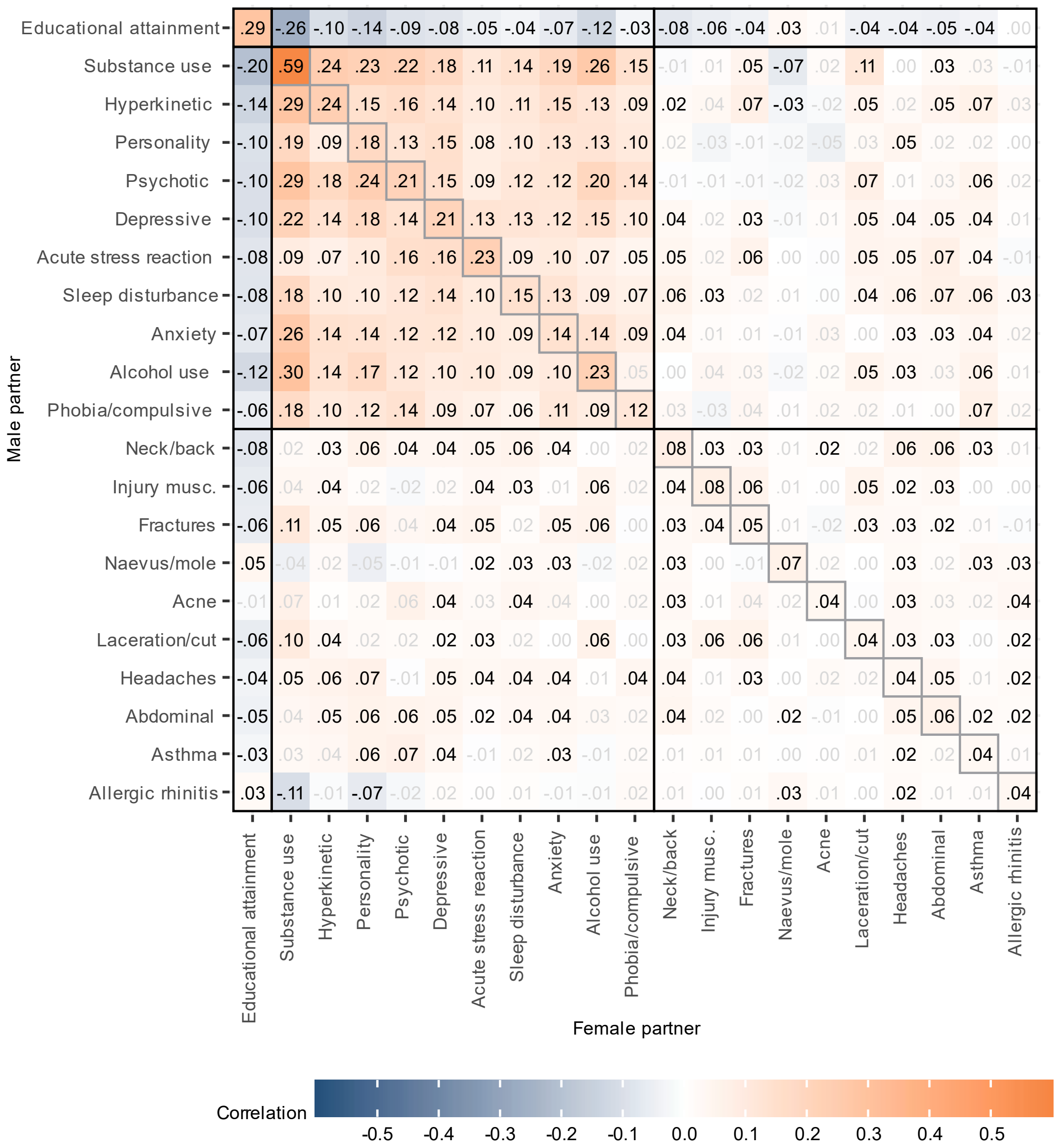

Supplemental Figure S8. Cross-sectional partner correlations adjusted for grade point average. Within and across-trait partner correlations for 10 mental health conditions, and 10 somatic health conditions, measured cross sectionally in 2015-2019 (n=93,963 couples). Adjusted for age and grade point average attainment. We tested whether the correlations differ from zero using two-sided z-tests based on the estimated correlations and their standard errors provided by OpenMx. Significant correlations ($p < 0.05$ after Benjamini-Hochberg adjustment) are shown in black. Exact p-values are provided in the Source Data file.

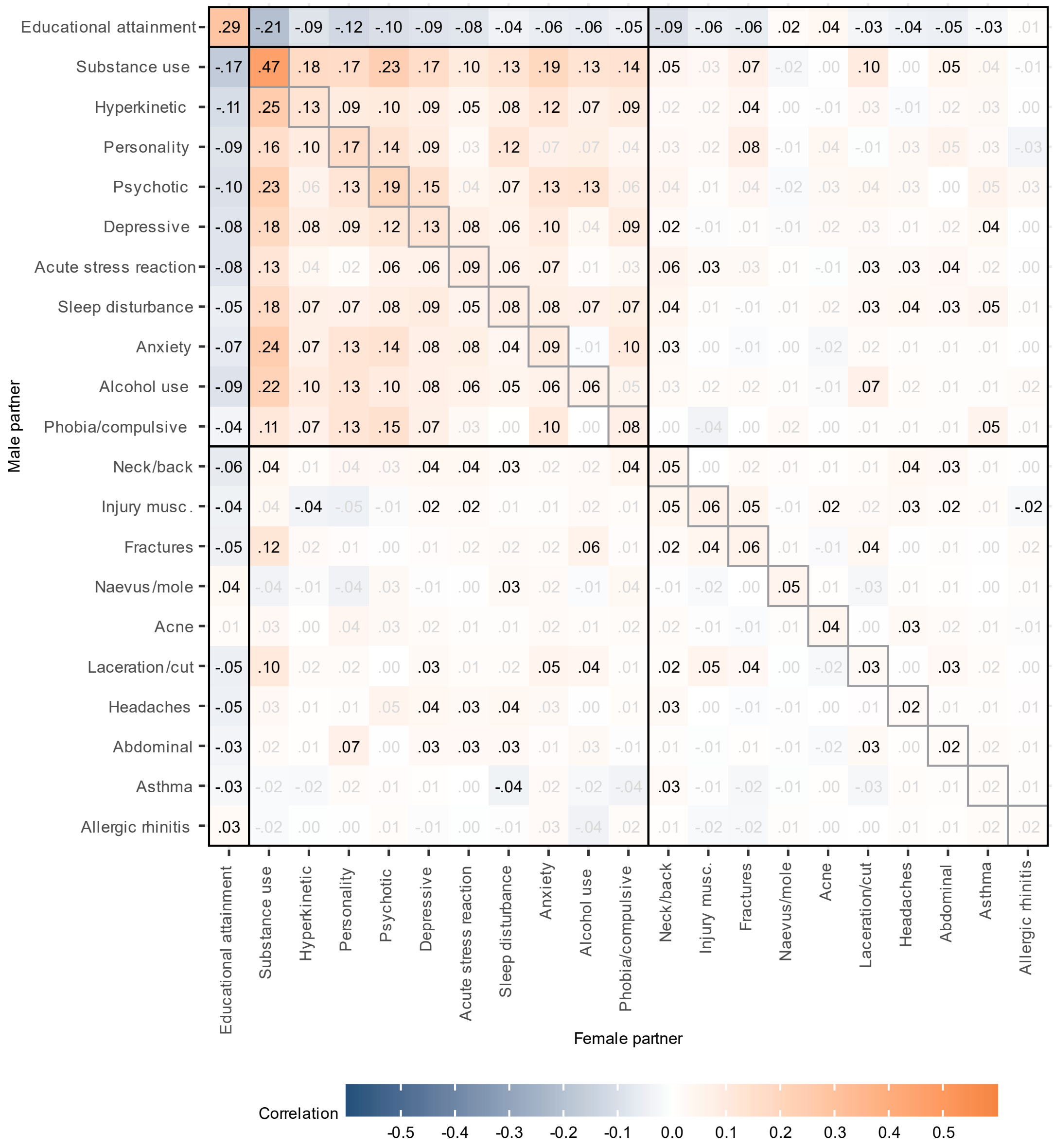

Supplemental Figure S9. Cross-sectional partner correlations adjusted for educational attainment. Within and across-trait partner correlations for 10 mental health conditions, and 10 somatic health conditions, measured cross sectionally in 2015-2019 (n=93,963 couples). Adjusted for age and educational attainment. We tested whether the correlations differ from zero using two-sided z-tests based on the estimated correlations and their standard errors provided by OpenMx. Significant correlations ($p < 0.05$ after Benjamini-Hochberg adjustment) are shown in black. Exact p-values are provided in the Source Data file.

```
library(tidyverse)
N = 10000
IIF = function(m,a,r,x,n,q,e) {
  rpartner = m*a*a+m*x*x+2*x*a+n*n+q*q
  rsibling = a*a*r+e+q*q
  rinlaw = (a*m+x)*r*a+q*q
  rinlaw/(rpartner*rsibling)
}

d = tibble(
  m = runif(N),
  a = runif(N),
  r = runif(N),
  x = 0,
  n = 0,
  q = 0,
  re = runif(N),
  e = re*(1-a^2),
  iif = IIF(m,a,r,x,n,q,e)
)
d %>% filter(iif < 1) %>% count(re > r)
```

Supplemental Script S1. R script illustrating of how to simulate the in-law inflation factor (IIF) under various forms of assortment. The current scenario demonstrates indirect assortment and that IIF is above 1.00 except in the implausible cases where $r_e < r_s$.

```r
library(polycor)

# dichotomized normally distributed variables
n = 10000
vc = rnorm(n)
va = vc+rnorm(n)
vb = vc+rnorm(n)

va_dich = ifelse(va<2,0,1)
vb_dich = ifelse(vb<2,0,1)

# pearson correlation between original variables
cor(va,vb)

# pearson correlation between dichotomized variables (underestimated)
cor(va_dich, vb_dich)

# polychoric correlation between dichotomized variables (correct)
polychor(va_dich,vb_dich)


# dichotomized skewed variables
n = 100000
vc = rnorm(n)
va = (vc+rnorm(n))^2
vb = (vc+rnorm(n))^2

hist(va)

va_dich = ifelse(va<5,0,1)
vb_dich = ifelse(vb<5,0,1)

# pearson correlation between original variables
cor(va,vb)

# pearson correlation between dichotomized variables
cor(va_dich, vb_dich)

# polychoric correlation between dichotomized variables (overestimated)
polychor(va_dich,vb_dich)
```

Supplemental Script S2. R script estimating tetrachoric correlations for variables with underlying normal or skewed distributions. Tetrachoric correlations get overestimated when based on non-normal variables. Written for R.

```
library(tidyverse)
library(polycor)

(randvar=runif(1))

# normally distributed variables

n = 100000
common1 = rnorm(n)
person_i   = common1 + rnorm(n)*randvar      # index
person_ip  = common1 + rnorm(n)*randvar      # index' partner
person_ips = person_ip/2 + rnorm(n)*randvar  # index' partner's sibling
(inlaw)

# correlation matrix
cor(tibble(person_i, person_ip, person_ips))

# observed correlations between indirectly associated variables (e.g.
between in-laws)
cor(person_i, person_ips)

# observed product of correlations (matches the expectation)
cor(person_i, person_ip)*cor(person_ip, person_ips)

# skewed variables
person_i2 = person_i^2
person_ip2 = person_ip^2
person_ips2 = person_ips^2

# correlation matrix
cor(tibble(person_i2, person_ip2, person_ips2))

# observed correlations between indirectly associated variables (e.g.
between in-laws)
cor(person_i2, person_ips2)

# observed product of correlations (still matches the expectation)
cor(person_i2, person_ip2)*cor(person_ip2, person_ips2)

# dichotomized skewed variables
threshold=3
person_i2_dich = ifelse(person_i2<threshold,0,1)
person_ip2_dich = ifelse(person_ip2<threshold,0,1)
person_ips2_dich = ifelse(person_ips2<threshold,0,1)

# correlation matrix
cor(tibble(person_i2_dich, person_ip2_dich, person_ips2_dich))

# observed pearson correlations between indirectly associated variables
(e.g. between in-laws)
cor(person_i2_dich, person_ips2_dich)

# observed product of pearson correlations (lower than the expectation)
cor(person_i2_dich, person_ip2_dich)*cor(person_ip2_dich, person_ips2_dich)

# observed polychoric correlations between indirectly associated variables
(e.g. between in-laws)
polychor(person_i2_dich, person_ips2_dich)

# observed product of polychoric correlations (matches the expectation
again)
polychor(person_i2_dich, person_ip2_dich)*polychor(person_ip2_dich,
person_ips2_dich)
```

Supplemental Script S3. R script estimating the product of tetrachoric correlations for skewed variables. This script shows that if the product of two Pearson correlations matches with a third correlation, this is also the case for tetrachoric correlations after dichotomization, even if the underlying variables are skewed. Written for R.
