# Supplementary material for: Non-random mating patterns within and across education and mental and somatic health
Source: Nat Commun. 2024 Dec 3;15:10505. doi: 10.1038/s41467-024-54966-9 (PMC11615381; doi:10.1038/s41467-024-54966-9)
Supplement: Supplementary file 2 — Reporting Summary [file 41467_2024_54966_MOESM2_ESM.pdf]

nature portfolio

Corresponding author(s): Fartein Ask Torvik

Last updated by author(s): Nov 5, 2024

Reporting Summary

Nature Portfolio wishes to improve the reproducibility of the work that we publish. This form provides structure for consistency and transparency in reporting. For further information on Nature Portfolio policies, see our Editorial Policies and the Editorial Policy Checklist.

Statistics

For all statistical analyses, confirm that the following items are present in the figure legend, table legend, main text, or Methods section.

n/a	Confirmed	
☐	☒	The exact sample size (*n*) for each experimental group/condition, given as a discrete number and unit of measurement
☐	☒	A statement on whether measurements were taken from distinct samples or whether the same sample was measured repeatedly
☐	☒	The statistical test(s) used AND whether they are one- or two-sided<br>*Only common tests should be described solely by name; describe more complex techniques in the Methods section.*
☐	☒	A description of all covariates tested
☐	☒	A description of any assumptions or corrections, such as tests of normality and adjustment for multiple comparisons
☐	☒	A full description of the statistical parameters including central tendency (e.g. means) or other basic estimates (e.g. regression coefficient) AND variation (e.g. standard deviation) or associated estimates of uncertainty (e.g. confidence intervals)
☐	☒	For null hypothesis testing, the test statistic (e.g. *F*, *t*, *r*) with confidence intervals, effect sizes, degrees of freedom and *P* value noted<br>*Give P values as exact values whenever suitable.*
☒	☐	For Bayesian analysis, information on the choice of priors and Markov chain Monte Carlo settings
☒	☐	For hierarchical and complex designs, identification of the appropriate level for tests and full reporting of outcomes
☐	☒	Estimates of effect sizes (e.g. Cohen's *d*, Pearson's *r*), indicating how they were calculated

Our web collection on statistics for biologists contains articles on many of the points above.

Software and code

Policy information about availability of computer code

Data collection	No software was used for data collection (we used existing data)
Data analysis	We used R 4.1.3 and OpenMx 2.21.8

For manuscripts utilizing custom algorithms or software that are central to the research but not yet described in published literature, software must be made available to editors and reviewers. We strongly encourage code deposition in a community repository (e.g. GitHub). See the Nature Portfolio guidelines for submitting code & software for further information.

Data

Policy information about availability of data

All manuscripts must include a data availability statement. This statement should provide the following information, where applicable:

- Accession codes, unique identifiers, or web links for publicly available datasets
- A description of any restrictions on data availability
- For clinical datasets or third party data, please ensure that the statement adheres to our policy

The data for this study encompass educational outcomes and primary care records for entire cohorts of the Norwegian population. The raw data are protected and are not available due to data privacy laws. Researchers can access the data by application to the Regional Committees for Medical and Health Research Ethics and the data owners (Statistics Norway and the Norwegian Directorate of Health). The authors cannot share these data with other researchers due to the sensitive

nature and potential for identification. However, other researchers can contact the authors if they have questions concerning the data or overlapping research projects.

Research involving human participants, their data, or biological material

Policy information about studies with human participants or human data. See also policy information about sex, gender (identity/presentation), and sexual orientation and race, ethnicity and racism.

Reporting on sex and gender	The data consisted of 93,963 men and 93,963 women in opposite sex couples, as recoded in the Norwegian Population Register. We used sex as a dichotmous variable. The analyses concern composition of opposite-sex couples and the results are reported separately by sex, except for couple-level results.
Reporting on race, ethnicity, or other socially relevant groupings	The Norwegian population is mostly of European ancestry, potentially limiting generalizability to other populations. We do not have data on ethnicity. We used data on educational attainment as recoded in the Norwegian Educational Database. Some of the analyses were adjusted for educational attainment of both partners by treating these variables as linear covariates.
Population characteristics	This population based study included 187,926 individuals in opposite sex-couples who became parents for the first time between 2016 and 2020, as identified in the Norwegian Population Register. This sample was chosen to observe health without potential participation bias. We included only couples who were both registered as living in Norway for the 10 to 5 years prior to the child's birth to be able to observe health. Women were on average 19.61 and men 21.96 years old at the start of the observational period.
Recruitment	All residents of Norway are assigned a primary-care physician (PC). To receive reimbursemets, PCPs bill the Norwegian Health Economics Administration, sending diagnosis or reason for the visit.
Ethics oversight	The study was approved by The Regional Committees for Medical and Health Research Ethics, Southern and Eastern Norway (project #2018/434).

Note that full information on the approval of the study protocol must also be provided in the manuscript.

Field-specific reporting

Please select the one below that is the best fit for your research. If you are not sure, read the appropriate sections before making your selection.

☐ Life sciences ☒ Behavioural & social sciences ☐ Ecological, evolutionary & environmental sciences

For a reference copy of the document with all sections, see nature.com/documents/nr-reporting-summary-flat.pdf

Behavioural & social sciences study design

All studies must disclose on these points even when the disclosure is negative.

Study description	Nationwide population reigstry study. The data are quantitative.
Research sample	This population based study included 187,926 individuals in opposite sex-couples who became parents for the first time between 2016 and 2020, as identified in the Norwegian Population Register. This sample was chosen to observe health without potential participation bias. We included only couples who were both registered as living in Norway for the 10 to 5 years prior to the child's birth to be able to observe health. Women were on average 19.61 and men 21.96 years old at the start of the observational period.
Sampling strategy	We used data for all individuals recorded in Norwegian administrative data. We did not conduct sample size calculation because the size of this population was fixed.
Data collection	As our study is based on administrative data, the researchers were not present during data collection. We used diagnoses coded according to ICPC-2 by primary care physicians who were not aware of the study hypotheses.
Timing	Health data was collected 2006-2019, births until 2020, and educational attainment until 2022.
Data exclusions	Same-sex couples were not included. Possible differences in data quality, mate preferences, and fertility imply that same-sex couples warrant separate studies. Also, we only used data for individuals recorded as settled in Norway, and not, for example, recent immigrants, paperless individuals, or tourists.
Non-participation	No participants dropped out or declined participation.
Randomization	Randomization was not relevant for this study.

Reporting for specific materials, systems and methods

We require information from authors about some types of materials, experimental systems and methods used in many studies. Here, indicate whether each material, system or method listed is relevant to your study. If you are not sure if a list item applies to your research, read the appropriate section before selecting a response.

Materials & experimental systems

n/a	Involved in the study
☒	☐ Antibodies
☒	☐ Eukaryotic cell lines
☒	☐ Palaeontology and archaeology
☒	☐ Animals and other organisms
☒	☐ Clinical data
☒	☐ Dual use research of concern
☒	☐ Plants

Methods

n/a	Involved in the study
☒	☐ ChIP-seq
☒	☐ Flow cytometry
☒	☐ MRI-based neuroimaging

Plants

Seed stocks	*Report on the source of all seed stocks or other plant material used. If applicable, state the seed stock centre and catalogue number. If plant specimens were collected from the field, describe the collection location, date and sampling procedures.*
Novel plant genotypes	*Describe the methods by which all novel plant genotypes were produced. This includes those generated by transgenic approaches, gene editing, chemical/radiation-based mutagenesis and hybridization. For transgenic lines, describe the transformation method, the number of independent lines analyzed and the generation upon which experiments were performed. For gene-edited lines, describe the editor used, the endogenous sequence targeted for editing, the targeting guide RNA sequence (if applicable) and how the editor was applied.*
Authentication	*Describe any authentication procedures for each seed stock used or novel genotype generated. Describe any experiments used to assess the effect of a mutation and, where applicable, how potential secondary effects (e.g. second site T-DNA insertions, mosiacism, off-target gene editing) were examined.*
